# Pattern recognition receptors in Crustacea: immunological roles under environmental stress

**DOI:** 10.3389/fimmu.2024.1474512

**Published:** 2024-11-14

**Authors:** Jesús Luis Betancourt, Tania Rodríguez-Ramos, Brian Dixon

**Affiliations:** Department of Biology, University of Waterloo, Waterloo, ON, Canada

**Keywords:** aquaculture, crustaceans, innate immunity, pattern recognition receptors, antimicrobial peptides, environmental stressors

## Abstract

Innate immunity is the first line of defense against infections and the only known available strategy for invertebrates. Crustaceans, being mostly aquatic invertebrates, are constantly exposed to potential pathogens in the surrounding water. Their immune system abolishes most microbes that enter and are recognized as a threat. However, the stress produced by high population densities and abiotic changes, in aquaculture, disrupts the host-pathogen balance, leading to severe economic losses in this industry. Consequently, crustacean immunology has become a prime area of research where significant progress has been made. This review provides our current understanding of the key pattern recognition receptors in crustaceans, with special focus on Decapoda, and their roles in triggering an immune response. We discuss recent developments in the field of signal transduction pathways such as Toll-like receptors (TLRs) and the immune deficiency (IMD) pathway, and examine the role of antimicrobial peptides (AMPs) in pathogen defense. Additionally, we analyze how environmental stressors—such as temperature fluctuations, ammonia levels, and pollution—impact immune responses and increase susceptibility to diseases. Finally, we highlight future research directions, emphasizing the need to explore the interactions between environmental stressors and immune signaling pathways and to develop strategies to enhance immune responses in crustaceans within aquaculture settings. Altogether, these advancements deepen our understanding of pathogen recognition in invertebrates and the specific defense mechanisms employed by crustaceans, particularly in response to infections triggered by pathogens under abiotic stressors.

## 1 Introduction

Innate immunity is the first line of defense against infections and the type of immunity available for invertebrates. Acquired immunity is believed to have been established about 500 million years ago with the arrival of the early jawed vertebrates and jawless fishes (1, 2). Nevertheless, invertebrates are a very successful group that have colonized most habitats on Earth and can resist numerous microbial infections, ensuring their survival (3).

Crustaceans are an ancient and prosperous group of invertebrates, comprising more than 67,000 described living species (4), many of which are used for human consumption and as consequence have an extraordinary economic importance worldwide (5). Over the last two decades, crustacean aquaculture has grown dramatically, becoming one of the pivotal sectors of the aquaculture industry. Currently, due its resistance to abiotic changes and diseases, the Pacific white shrimp, *Litopenaeus* (*Penaeus) vannamei*, is the most cultivated shellfish species worldwide, with an increase in production from 0.15 million tonnes in 2000 to 5.8 million tonnes in 2020 (5).

Despite this rise and expansion through the years, aquaculture faces considerable loses due to disease outbreaks caused by parasitic, viral, and bacterial pathogens, representing approximately USD 50 billion annually, more than 6 billion for fish and 43 billion from shrimp in 2021 (6, 7). These outbreaks are usually triggered by environmental stressors such as extreme temperature fluctuations and contaminants like plastics and pharmaceuticals, which impose metabolic costs to maintain homeostasis, weaken immune defenses, and increase disease vulnerability (8). In this context, the primary pathogens affecting cultured crustaceans are viruses, for example White Spot Syndrome Virus (WSSV), and bacteria of the genus *Vibrio* such as *Vibrio harveyi*, and *Vibrio parahaemolyticus* which cause Acute Hepatopancreatic Necrosis Disease (AHPND) (9). Giving this background, farmers are overusing and misusing antibiotics as a prophylactic treatment to avoid infectious diseases, which has led to antibiotic resistance in human and farm animal pathogens (10). Therefore, crustacean immunology has become a prime area of research looking for the development of new and effective therapies and husbandry practices that can improve aquaculture species’ health and responses to diseases.

In light of these insights, this review seeks to provide an overview of our current understanding of crustacean pattern recognition receptors, emphasizing their features, signaling pathways and immune responses triggered. Moreover, we highlight the key environmental stressors affecting crustacean aquaculture, underscoring their implications for crustacean health and their connections to signaling pathways.

## 2 Key components of Crustaceans immune system

Aquatic crustaceans are constantly exposed to opportunistic and obligate pathogens in the surrounding water, yet their immune system abolishes most microbes that successfully manage to breach their physical barriers (11). These environmental interfaces consist of a strong exoskeleton, a digestive system protected by physical and chemical mechanisms, and a robust epithelial cell layer combined with a cutin surrounding the gills, block the entry of most microbes (12).

However, when pathogens successfully break these barriers, they encounter a sophisticated immune system composed of cellular and humoral responses designed to neutralize invasive agents. The cell-mediated responses include processes such as phagocytosis, nodule and capsule formation, and hemocyte degranulation. The latter serves as a crucial starting point, since it triggers the release of antimicrobial peptides (AMPs) and clotting factors, which subsequently contribute to the processes of encapsulation and nodule formation (13). Moreover, this secretion of immune components constitutes a vital link between cellular and humoral immunity in crustaceans (12).

Humoral responses comprise mechanisms such as pathogen recognition, hemolymph coagulation, activation of the prophenoloxidase-activating system (proPO system), and the production and release of AMPs and antiviral factors [see recent review by (14)]. Upon recognition of pathogen-associated molecular patterns (PAMPs), immune cascades are triggered, leading to hemolymph coagulation and melanin production, which help contain and destroy pathogens [see also (14–16)]. The proPO system also plays a critical role by promoting the melanization of pathogens, thus enhancing immune protection (17). Additionally, AMPs, lysozymes, and lectins provide broad-spectrum antimicrobial activity, safeguarding crustaceans from various infectious agents (18).

## 3 Pattern recognition proteins

As part of the immune system, PRPs are a group of germ-line-encoded proteins in charge of recognition of PAMPs and triggering immune reactions. Most commonly encountered PAMPs are polysaccharides and glycoproteins such as lipopolysaccharide (LPS) from Gram-negative (Gram^-^) bacteria, peptidoglycan (PGN) and lipoteichoic acid (LTA) from Gram-positive (Gram^+^) bacteria, and β-glucans (βG) from fungal cells, all of which are usually exposed on the surface of microbes (19). Furthermore, intracellular components like polynucleotides such as unmethylated CpG DNA, single-strand and double-strand RNA can also act as PAMPs (20). In crustaceans, a suite of PRPs have been categorized depending on their binding properties and the immune reactions triggered. These proteins are grouped in families such as β-1,3-glucanase-related proteins (BGRP), β-1,3-glucan-binding proteins (BGBP), lectins, scavenger receptors (SCR), thioester-containing proteins (TEP), and Down syndrome cell adhesion molecule (DSCAM) proteins (21). Herein an overview is provided, emphasizing the most relevant PRPs and their related immune functions (**Table 1**).

**Table 1:** Principal pattern recognition proteins in crustaceans.

Pattern recognition proteins	Ligands	Species	References
β-1,3-glucanase-related proteins (BGRP)	Lipopolysaccharide β-glucan	*Litopenaeus vannamei* *Penaeus monodon* *Marsupenaeus japonicus* *Fenneropenaeus chinensis* *Procambarus clarkii*	(22) (23) (24) (25) (26)
β-1,3-glucan-binding protein (BGBP)	β-1,3-glucan	*Litopenaeus vannamei* *Penaeus semisulcatus* *Penaeus californiensis* *Penaeus leniusculus* *Penaeus stylirostris*	(27, 28) (29) (28) (30) (28)
C-type lectins	Carbohydrates Lipopolysaccharide β-glucan Lipoteichoic acid WSSV proteins	*Litopenaeus vannamei* *Eriocheir sinensis* *Litopenaeus setiferus* *Marsupenaeus japonicus* *Scylla paramamosain* *Fenneropenaeus chinensis* *Penaeus monodon*	(31–33) (34) (32) (35) (36) (37)
Galectin	Carbohydrates Lipopolysaccharide Lipoteichoic acid	*Marsupenaeus japonicus* *Litopenaeus vannamei* *Penaeus monodon* *Eriocheir sinensis*	(15)
Fibrinogen-related proteins (FREP)	Carbohydrates Lipopolysaccharide WSSV proteins	*Marsupenaeus japonicus* *Penaeus monodon* *Litopenaeus vannamei* *Macrobrachium rosenbergii* *Macrobrachium nipponense* *Tachypleus tridentatus* *Procambarus clarkii*	(15)
Thioester-containing proteins (TEP)	PAMPs	*Marsupenaeus japonicus* *Penaeus monodon* *Litopenaeus vannamei* *Fenneropenaeus chinensis* *Penaeus leniusculus*	(12, 15)
Scavenger receptor	PAMPs Carotenoid	*Litopenaeus vannamei* *Penaeus monodon* *Scylla paramamosain* *Marsupenaeus japonicus*	(38) (39) (40)
Down syndrome cell adhesion molecule (DSCAM)	PAMPs	*Penaeus monodon* *Litopenaeus vannamei* *Pacisfastacus leniusculus* *Cherax quadricarinatus* *Eriocher sinensis*	(41, 42)

β-1,3-glucanase-related proteins (BGRPs), previously named as lipopolysaccharide and β-1,3-glucan binding proteins (LGBP), are a representative PRP family described for insects and crustaceans (43). A conserved sequence region similar to the β-glucanases (glucanase like domain, GLU domain) from bacteria is the prominent characteristic of this family (43, 44). These proteins mostly recognize LPS from Gram^-^ bacteria and βG from fungal cells (43). Its expression is detected almost specifically in hemocytes and hepatopancreas of *L. vannamei* (22); however, in *Penaeus monodon* (23) and *Marsupenaeus japonicus* (24) it was exclusively detected in hemocytes. Northern blot studies in *Fenneropenaeus chinensis* also revealed the presence of BGRP only in the hemocytes (25); nevertheless, RT-qPCR analysis showed the existence of another BGRP isoform in the hemocytes and hepatopancreas (45). Thus, BGRP is expressed in hemocytes and/or hepatopancreas of crustaceans.

Crustacean BGRPs present two cell adhesion motifs (Arg-Gly-Asp (RGD) motif), absent in insect counterparts, which are important for the interaction with integrin receptors on the cell surface (12). This protein interaction might trigger the degranulation of granular and semi-granular hemocytes and the release of its immune components such as the proPO activating system (46). In this sense, Chai et al. (26) by a combination of *in vivo* and *in vitro* studies in *Procambarus clarkii* provided evidence supporting the relation between high level of BGRP expression and enhanced PO activation in response to a challenge with *Aeromonas hydrophila*. Furthermore, augmented PO activation was reported in hemocyte lysate supernatant incubated with LPS/βG and BGRP, in contrast with only LPS/βG incubation. The authors argue that the increase or acceleration of the PO activation suggests a key role of this PRP as an upstream sensor for the whole system (26).

The β-1,3-glucan binding protein (βGBP) is a type of lipoglycoprotein synthesized by the hepatopancreas and constitutively secreted into the hemolymph (47, 48). This protein acts as PRP by recognizing β-1,3-glucans and inducing hemocytes degranulation through its RGD and/or RGE (Arg-Gly-Glu, (RGE) motif) motifs; thus, activating the pro-PO system. The formation of glucan-βGBP complex is required to induce a conformational change that allows the interaction with the hemocyte receptors (27, 49). Moreover, βGBP is involved in agglutination of fungal cells (50) and enhances phagocytosis by hemocytes (51, 52). High density lipoprotein (HDL) is the main non-sex-specific lipoprotein involved in the transport of lipids in crustaceans (53, 54). Interestingly, the βGBP and HDL are the same protein as has been demonstrated in *L. vannamei* (27, 28), *Penaeus semisulcatus* (29), *Penaeus californiensis* (28), *Penaeus leniusculus* (30) and *Penaeus stylirostris* (28), suggesting a close relationship between the ability to respond to PAMPs and the transport of essential lipids provided by the diet (28).

Agglutination is one of the key humoral responses against invading microorganisms. Lectins are the most well-known agglutinating factors, able to recognize several PAMPs as well as damage-associated molecular patterns (DAMPs) (55). One of the main characteristics of these proteins is a carbohydrate recognition domain (CRD), which confers different carbohydrate binding affinities based on their structure. Thus, lectins can be clustered into groups based on their ligand specificity (55). For instance, in crustaceans a broad range of lectin specificities that cluster into at least seven types including C-type, L-type, M-type, P-type, fibrinogen-like proteins (FREPs), galectins, and calnexin/calreticulin (53) have been described. C-type lectins (CTLs) are the most diverse and well-studied group (37). Crustacean CTLs are named after their Ca^2+^ dependent activity; and can present one or more CRD, but a single CRD being the most usual (12).

Crustacean CTLs [Reviewed by (28, 53, 54)] have been characterized as mainly secretory proteins with higher expression in hemocytes and hepatopancreas compared to other tissues (15). Besides agglutination (31, 32, 34, 35, 56–58), these proteins elicit several other immune responses such as phagocytosis (57, 59), encapsulation (34), respiratory burst (32), proPO system activation (58), and antiviral responses (56, 60). Overall, the activity and expression of CTLs enhances during Gram^-^ (*Pseudomonas spp* and *Vibrio* spp) and Gram^+^ (*Staphylococcus aureus*) bacterial, and fungal (*Aspergillus niger*) infections (33). Likewise, studies show that CTLs can bind to WSSV’s envelope proteins and trigger the expression of AMPs through intracellular signaling pathways, thus facilitating the eradication of this virus (40, 57, 58).

DSCAMs are portrayed as hypervariable PRPs which can generate several isoforms by alternative splicing of variable exons from a single-locus gene [Reviewed by (41, 42, 61)]. Thus, DSCAM have been proposed as mediators of a version of challenge-specific protection (62). Nevertheless, almost two decades after their discovery, there is still a lack of definitive information about DSCAM in long-term immune modulation after pathogen exposure or whether DSCAM plays a role in specificity upon secondary encounters (61). Therefore, the notion that these proteins act as an immunological effector enhancing immune memory lacks solid empirical support, and our understanding of DSCAM’s regulatory roles in the immunity of crustaceans is still incomplete. Further research needs to address long-term regulation of DSCAM after repeated pathogen exposure; moreover, it remains unclear how specific isoforms are modulated and what their role is in immune memory (61). Elucidating these regulatory mechanisms is crucial for understanding DSCAM’s role in immunity in crustaceans.

## 4 Cell-associated PRPs

Most cell types express PRPs and consequently can participate in innate immune responses. Cell-associated PRPs are linked to intracellular signal transduction pathways that trigger several cellular responses, including the production of AMPs. The main PRP-signaling pathways present in invertebrates are Toll like-receptors (TLRs), immune deficiency (IMD) and Janus kinase-signal transducer and activator of transcription (JAK-STAT) signaling pathways (11, 63–66). Currently, several research groups have focused their attention on testing and characterizing the presence of these receptors in crustacean species.

## 5 TLRs in Crustaceans

To date, TLRs are the most extensively studied receptors in crustaceans, mainly in decapod species (**Table 2**). Originally discovered in *Drosophila melanogaster* (105) but described across a wide range of invertebrate and vertebrate species (106, 107), TLRs are type I integral membrane glycoproteins that contain three structural domains: a leucine-rich repeats (LRRs) flanked by characteristic cysteine-rich motifs, a transmembrane domain and a cytoplasmic Toll/interleukin-1 receptor (TIR) domain. The LRR motif is involved in PAMPs and DAMPs recognition, whereas the TIR domain interacts with signal transduction adaptors and initiates signaling (108, 109). This signaling cascade in invertebrates is based on the canonical mechanism of *D. melanogaster* Toll1 (DmToll), where DmToll is not a direct PRP, unlike most TLRs in mammals (110). In this case, soluble PRPs such as Gram^-^ bacteria-binding protein (GNBP), peptidoglycan recognition protein (PGRP) and persephone perform PAMP recognition and trigger a serine protease cascade to cleave pro-Spätzle to Spätzle (Spz), an active ligand for the DmToll receptor activation (111–117). Once DmToll receptors are activated, their TIR domains recruit the adaptor, myeloid differentiation factor 88 (MyD88) followed by the recruitment of the second adaptor Tube and the protein kinase Pelle, to form the receptor complex. Pelle´s activation results in Cactus phosphorylation and degradation, freeing Dorsal and Dorsal-related immunity factor (DIF) (19, 118–121) to translocate into the nucleus to regulate the transcription of AMP genes (122–124). Moreover, this complex causes the recruitment of an E3 ubiquitin ligase (named Pellino) (125, 126) and the *Drosophila* homolog of tumor necrosis factor receptor-associated factor 6 (TRAF6), dTRAF2 (127).

**Table 2:** Components of crustaceans’ Toll signaling pathway.

Components	Species	References
Toll	*Litopenaeus vannamei*	(63, 67–70)
	*Marsupenaeus japonicus*	(71)
	*Penaeus monodon*	(72–74)
	*Procambarus clarkii*	(75–78)
	*Fenneropenaeus chinensis*	(79)
	*Macrobrachium rosenbergii*	(80, 81)
	*Sinopotamon henanense*	(82)
	*Portunus trituberculatus*	(83)
	*Scylla paramamosain*	(84)
	*Eriocheir sinensis*	(85)
	*Cherax quadricarinatus*	(86)
Spätzle	*Litopenaeus vannamei*	(67, 87)
	*Penaeus monodon*	(88)
	*Fenneropenaeus chinensis*	(89)
	*Marsupenaeus japonicus*	(90)
	*Macrobrachium rosenbergii*	(91)
MyD88	*Penaeus monodon*	(92)
	*Litopenaeus vannamei*	(93)
	*Fenneropenaeus chinensis*	(94)
Tube	*Litopenaeus vannamei*	(95, 96)
	*Penaeus monodon*	(97)
Pelle	*Litopenaeus vannamei*	(96)
Pellino	*Litopenaeus vannamei*	(98)
TRAF6	*Penaeus monodon*	(92)
	*Litopenaeus vannamei*	(99)
Cactus	*Litopenaeus vannamei*	(100)
Dorsal	*Macrobrachium rosenbergii*	(101)
	*Litopenaeus vannamei*	(102)
	*Fenneropenaeus chinensis*	(103)
	*Marsupenaeus japonicus*	(104)

**Figure 1:**
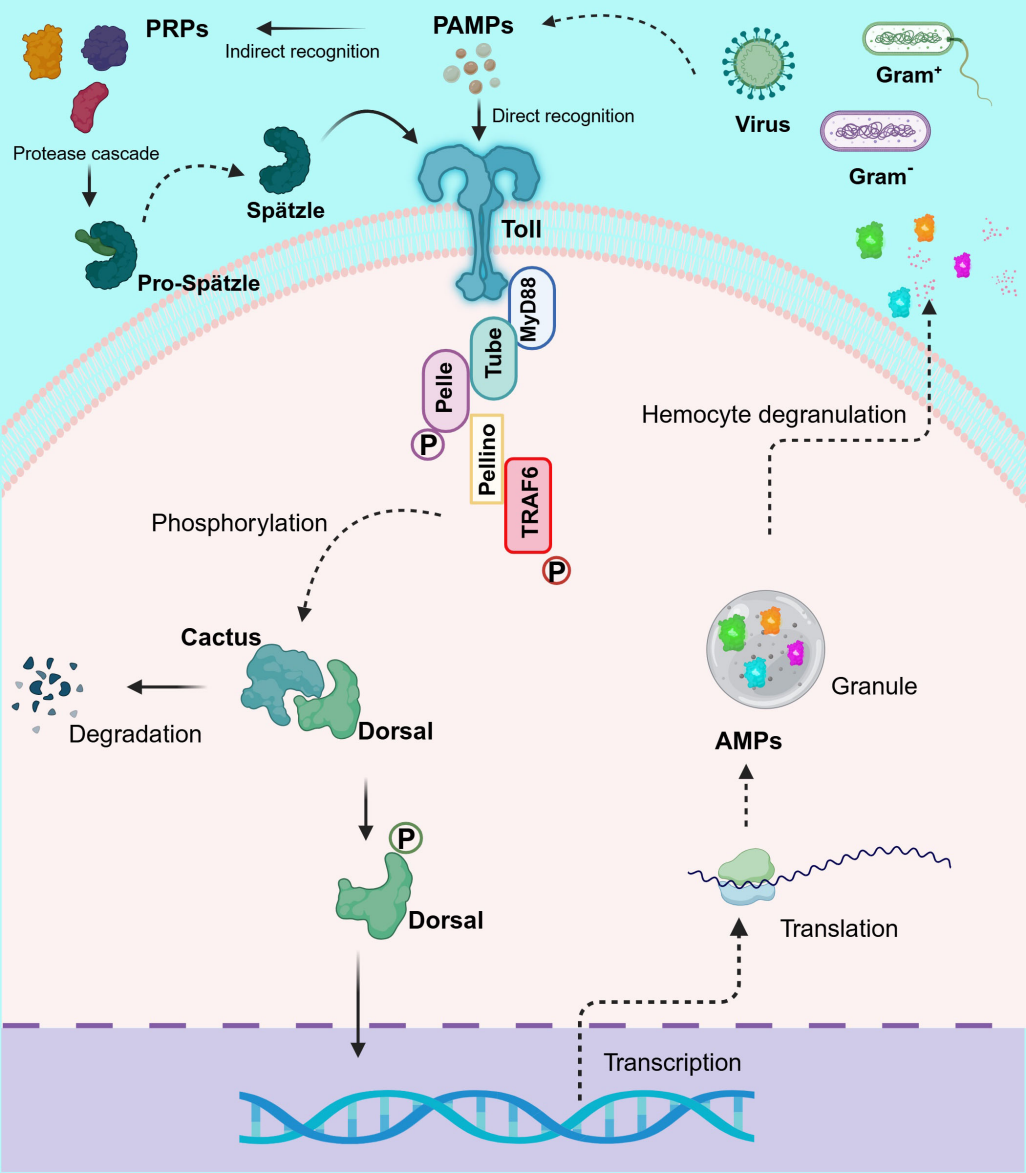
Schematic representation of the proposed Toll-like receptor (TLR) signaling pathway in shrimp. This diagram depicts the dual mechanism of TLR activation in crustaceans, highlighting both the cleavage of Spätzle (Spz) and direct recognition of pathogen-associated molecular patterns (PAMPs). Upon pathogen detection, TLRs activate signaling cascades by recruiting the adaptor protein MyD88, which assembles a complex with Tube and Pelle. This recruitment triggers the phosphorylation and degradation of Cactus, thereby releasing Dorsal (and/or Dorsal-related immunity factor, DIF) to translocate to the nucleus. Additionally, the complex engages Pellino and TRAF6, which further amplify the signaling process. The culmination of these events is the transcriptional activation of antimicrobial peptide (AMP) genes, critical for effective immune responses in shrimp. This pathway exhibits significant evolutionary conservation, reflecting similarities with TLR signaling in both *Drosophila* and mammals, while also showcasing unique adaptations in crustaceans. Created in BioRender. Betancourt Aguiar, J (2024). BioRender.com/y09c954.

In crustaceans, homologs for most components of the DmToll pathway have been described (**Table 2**), including Spz (67, 87–91), MyD88 (92–94), Tube (95–97), Pelle (96), Pellino (98), TRAF6 (92, 99), Cactus (100) and Dorsal (101–104, 128), suggesting a similar signaling pathway and immune functions. However, the activation and signaling mechanisms of TLRs in crustaceans (**Figure 1**) show significant differences from insects that make them more similar to mammals [see also (66)]. In line with this and based on the number of CF motifs (cysteine clusters at the C-terminal end of LRRs, LRRCT), TLRs can be classified into two types: single cysteine cluster or vertebrate-type (V-type), and multiple cysteine cluster or protostome-type (P-type) (129). The P-type has exclusively been identified in invertebrates, suggesting that it is an ancient form of TLR, whereas in all vertebrates and a few invertebrate exceptions, TLRs belong to the V-type (130–132). Most invertebrates TLRs belong to the P-type (63, 71, 72, 79–86) including all *Drosophila* Tolls, except DmToll9 (133, 134). Evidence suggests that P-type TLR cannot directly recognize PAMPs, in contrast to V-type TLR (129). Nevertheless, studies in *M. japonicus* (71, 134), *L. vannamei* (67, 133), *F. chinensis* (79), *Marsupenaeus rosenbergii* (80), and *P. monodon* (73) indicate that some crustacean TLRs can bind directly to PAMPs and activate signal transduction pathways, similar to TLRs in mammals. For instance, Sun et al. (134) discovered that three TLRs from *M. japonicus* can recognize Gram^+^ and Gram^-^ bacterial infection through direct binding of their LRR motifs to PGN and LPS, respectively. It is intriguing, in this study, that Gram^-^ bacterial infection could activate the TLR pathway despite the fact that DmToll mainly responds to Gram^+^ bacteria, fungi and some viruses (118); however, other reports in crustacean models (67, 68, 79, 80, 83) are in agreement with Sun et al. (134). Moreover, two TLR from *L. vannamei* can interact with CpG oligodeoxynucleotides (ODNs), suggesting their potential role in nucleic acid recognition, similar to TLR9 in mammals (67).

The structural basis for the interaction between crustacean TLRs and PAMPs seems to be explained by the presence of amino acid insertions around positions 10 or 15 of the LRRs ectodomain, as well as the existence of potential N-linked glycosylation sites (63, 71, 79, 80). In human TLRs, insertions following positions 10 and 15 in the LRR consensus sequence have been proposed to be essential for PAMP recognition (135). These insertions introduce flexibility into the concave surface (ligand-binding site) that could interact with PAMPs or accessory proteins such as MD-2 (135–137). In shrimp *L. vannamei* (63), *M. japonicus* (71), *F. chinensis* (79) and *M. rosenbergii* (80), insertions at position 10 were identified, in contrast to DmToll where none have been reported (138). On the other hand, TLR glycosylation plays a crucial role in receptor surface representation, trafficking, and ligand recognition, as supported by studies in human TLR2 (139) and TLR4 (140), TLR3 (141), and TLR5 (135, 142), respectively. Following this logic, sequence analysis in *M. japonicus* (71), *F. chinensis* (79) and *M. rosenbergii* (80) describe 16, 12 and 9 potential N-linked glycosylation sites, respectively, some of which could influence PAMP recognition. As argued by Bell et al. (135) structural variations at the LRRs motif, especially within the concave surface might provide the specificity needed for TLR responses to different pathogens.

Compelling evidence has shown that the function of P-type TLR structure is to provide the necessary framework for protein-protein interaction with Spz (107). However, it seems this is not their exclusive purpose, as it has been reported that these receptors can also directly recognize PAMP since, as mentioned before, this interaction could be influenced by amino acid insertions and glycosylation. The same phenomenon has been reported in other aquatic invertebrates like *Crassostrea gigas* (129) and *Hyriopsis cumingii* (143, 144).

Crustacean TLRs possess unique characteristics due to their ability to activate a defensive response either by direct recognition of PAMPs or Spz mediated activation. While the insect Toll network differs from mammalian TLRs in that a protease cascade links microbial detection by a soluble PRR to a downstream defensive response (145), the convergence of these two mechanisms could potentially increase protection against pathogens in these organisms. Direct recognition of PAMPs could elicit a more specific and targeted immune response giving rise to a more protective outcome. On the other hand, multiple PRPs can evolve to bind different PAMPs resulting in the activation of Spz, feeding information downstream to a single Toll receptor, thus providing the organisms with the ability to recognize a plethora of potential threats without the need for additional membrane receptors (145). Furthermore, a single pathogen contains thousands of PAMPs which can result in the activation of an even greater number of receptors in a potentially higher number of cells as compared to direct recognition by the membrane-bound Toll molecules (145, 146). This way a single pathogen (for example a bacterium) can amplify the number of cells participating in the subsequent immune response (145). Nevertheless, further studies of the structural characteristics of the LRR ectodomains are needed to draw more accurate conclusions on the nature of the interaction with PAMPs and the different specificities of TLR that facilitate recognition.

## 6 IMD pathway in Crustaceans

IMD is a death-domain containing protein encoded in a locus termed *immunodeficiency* (*imd*) (147). Mutation of this gene causes impaired production of AMPs and reduced survival to Gram^-^ bacterial infection in comparison to normal resistance to fungi and Gram^+^ bacteria (147, 148). Upon this discovery, first described in *Drosophila*, compelling evidence has shown that the IMD pathway is responsible for sensing diaminopimelic acid (DAP)-type PGN produced by Gram^-^ bacteria, as well as some Gram^+^ bacterial species such as *Listeria* and *Bacillus* (149). Whereas the TLRs pathway mainly responds to fungi and Gram^+^ bacteria with the Lys-type PGN (118). Mammals lack an IMD signaling cascade, but most components of tumor necrosis factor receptor (TNF-R) pathway broadly resemble the *Drosophila* IMD pathway nonetheless (150).

According to the current model in *Drosophila*, two receptors are implicated in specifically recognizing DAP-type PGN, peptidoglycan recognition protein-LC (PGRP-LC), which is located on the plasma membrane, and the intracellular PGRP-LE (149, 151). After binding to PGN these receptors likely dimerize or multimerize (152), and an intracellular signal is transmitted to the adaptor protein IMD (153). IMD interacts via its Death Domain (DD) with Fas-Associated protein with death domain (FADD), and FADD in turn recruits the caspase-8 homolog Death-related ced-3/Nedd2-like protein (DREDD) to the signaling complex via a homotypic Death-effector domain (DED) interaction (147, 149, 151, 154, 155). DD interaction is a key feature in cellular response to infection and all members of the DD superfamily promote inflammation or apoptosis which are essential to clear bacterial infection or prevent further viral replication (156). It has been proposed that DREDD is activated via ubiquitination mediated by inhibitor of apoptosis 2 (IAP2) a component of the ubiquitin machinery which functions as a E3-ubiquitin ligase. Activated DREDD cleaves IMD causing the exposure of a binding site for IAP2 which leads to K63-ubiquitinated IMD (155). K63-polyubiquitin chains of IMD recruit and activate the TGF-β activated kinase 1 (TAK1) via the ubiquitin-binding domain of its regulatory partner TAK1-associated binding protein (TAB2). Upon activation, the TAK1/TAB2 complex is responsible for the phosphorylation and activation of both the I-kappa B kinase complex (IKK) and the mitogen-activated protein kinase (MAPK) branches of the IMD pathway, which culminate in Relish and activator protein-1 (AP-1) activation respectively (154, 155, 157). Relish is a dual domain protein consisting in N-terminal Rel (or NF-kB) domain and a C-terminal ankyrin-repeat/I-kappa B-like domain. The N-terminal Rel domain is released by DREED-mediated cleavage and phosphorylated by IKK complex thereafter undergoes nuclear translocation and trigger the transcription of its target genes, such as AMPs (151, 158).

At the same time, TAK1 acts as a mitogen-activated protein kinase kinase kinase (MAPKKK) activating a pathway leading to c-Jun N-terminal kinase (JNK) and p38 MAPKs that results in the phosphorylation and activation of AP-1 transcription factors (such as c-Jun and c-Fos) ultimately triggering the promoters of a subset of immune-responsive target genes (149, 151). Interestingly, JNK and p38 MAPK are also known as stress-activating protein kinases (SAPKs), because of their ability to respond to a variety of cellular stresses such as oxidative stresses, UV irradiation, osmotic imbalance, heat shock, DNA-damaging agents, inflammatory cytokines and pathogen infection (159).

A growing number of orthologues of the main components of this *Drosophila* pathway have been isolated and cloned in crustaceans (**Table 3**), such as IMD (160–166), DREDD (GenBank number: XM_043017759), IAP2 (GenBank number: XM_043031423) TAK1 (167, 168), TAB2 (154), IKK complex (170), Relish (128, 163, 170–173), MKK4 (174, 175), MKK6 (176), MKK7 (177), JNK (178), c-Jun (179, 180), c-Fos (179), p38 (174, 181, 182), NF-kB repressing factor (NKRF) (183) and Akirin (184, 185), [see also (66)]. The first orthologue described was *L. vannamei* IMD (160, 161), which was also later identified in other crustacean species (**Table 3**).

**Table 3:** Components of crustaceans’ IMD signaling pathway. .

Components	Species	References
IMD	*Litopenaeus vannamei*	(160, 161)
	*Fenneropenaeus chinensis*	(162, 163)
	*Procambarus clarkii*	(162)
	*Macrobrachium nipponense*	(164)
	*Scylla paramamosain*	(165)
	*Portunus trituberculatus*	(166)
DREDD	*Marsupenaeus japonicus*	XM_043017759
IAP2	*Marsupenaeus japonicus*	XM_043031423
TAK1	*Litopenaeus vannamei*	(167, 168)
TAB2	*Litopenaeus vannamei*	(154)
TAB1	*Litopenaeus vannamei*	(169)
IKK complex	*Litopenaeus vannamei*	(170)
Relish	*Litopenaeus vannamei*	(128, 170, 171)
	*Fenneropenaeus chinensis*	(163)
	*Macrobrachium rosenbergii*	(172)
	*Penaeus monodon*	(173)
MKK4	*Fenneropenaeus chinensis*	(174)
	*Litopenaeus vannamei*	(175)
MKK6	*Litopenaeus vannamei*	(176)
MKK7	*Litopenaeus vannamei*	(177)
JNK	*Litopenaeus vannamei*	(178)
c-Jun	*Litopenaeus vannamei*	(179, 180)
c-Fos	*Litopenaeus vannamei*	(179)
p38	*Fenneropenaeus chinensis*	(174)
	*Litopenaeus vannamei*	(181, 182)
NF-kB repressing factor	*Litopenaeus vannamei*	(183)
Akirin	*Litopenaeus vannamei*	(184)
	*Marsupenaeus japonicus*	(185)

**Figure 2:**
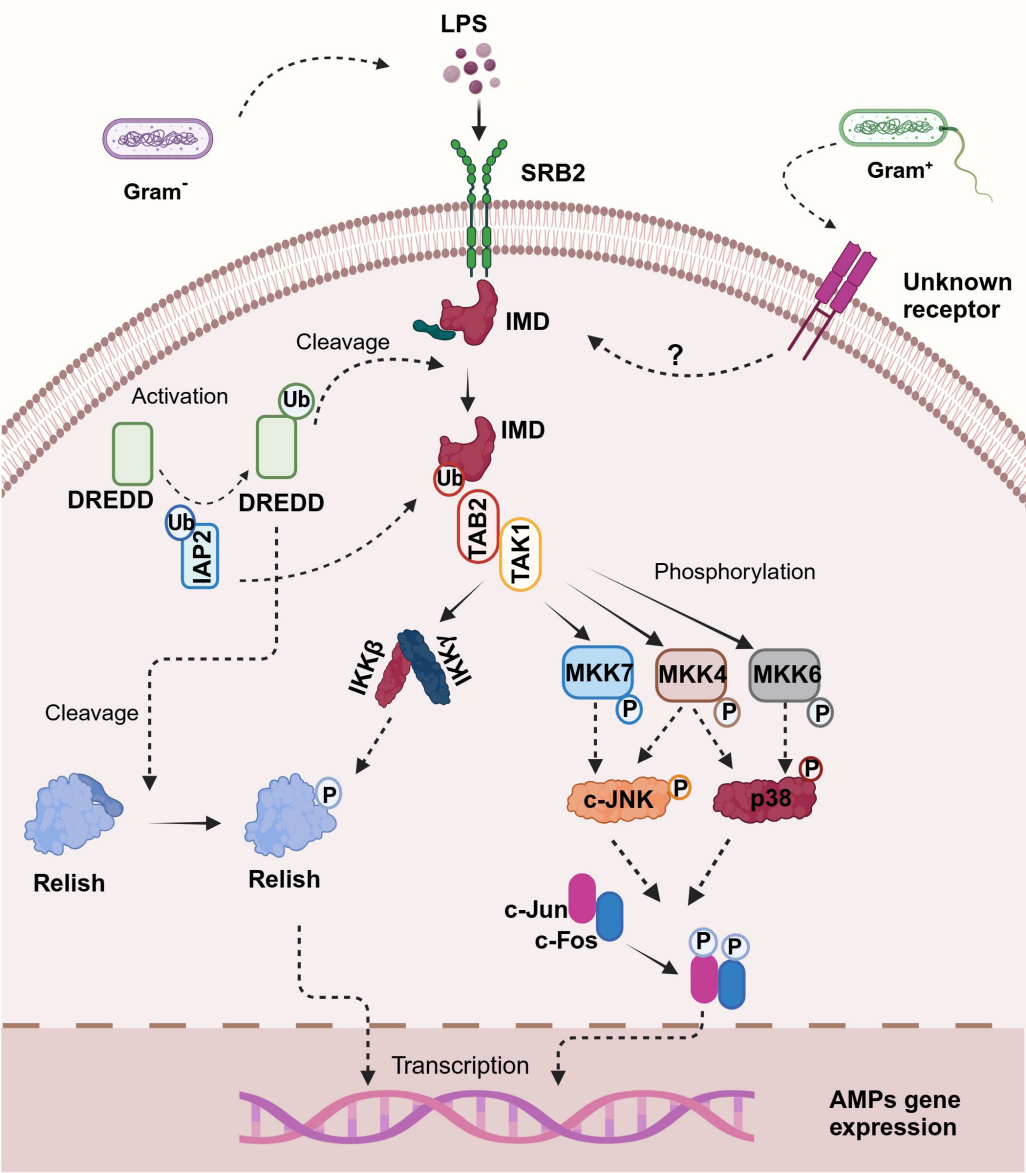
Illustration of the proposed immuno-deficiency (IMD) signaling pathway in shrimp. Upon binding of lipopolysaccharides (LPS) to the scavenger receptor class B2 (SRB2) — a proposed mechanism for crustaceans since no peptidoglycan recognition proteins (PGRPs) have been described—the pathway is initiated. This interaction leads to the recruitment and activation of DREDD via ubiquitination mediated by IAP2. Activated DREDD cleaves IMD, exposing a binding site for IAP2, which facilitates K63-ubiquitination of IMD. The K63-polyubiquitinated IMD recruits TAK1 through TAB2, activating the I-kappa B kinase (IKK) complex and mitogen-activated protein kinases (MAPK) pathways, specifically MKK4, MKK6, and MKK7. Notably, in crustaceans, p38 is activated by MKK4 and MKK6 through phosphorylation, paralleling human immune signaling mechanisms. This bifurcated signaling cascade culminates in the activation of Relish and AP-1 transcription factors, including c-Jun and c-Fos, which translocate to the nucleus and trigger the transcription of antimicrobial peptide (AMP) genes, orchestrating the immune response against bacterial infection. Created in BioRender. Betancourt Aguiar, J. (2024) BioRender.com/h56u584.

Even though the crustacean pathway resembles the *Drosophila*’s pathway in some aspects, there are some key differences (**Figure 2**). For instance, according to Li and coworkers (164) phylogenetic analysis indicated that there are two branches: receptor-interacting protein (RIP) from the vertebrate TNF-R pathway and IMD from invertebrates, which further bifurcated into the insect and crustacean sub-clusters. The correct clustering of these peptides in comparison to traditional taxonomy indicates evolution among IMD and RIP1 proteins (164). Furthermore, the IMD pathway displays significant discontinuities in the taxonomic distribution of key components, thus indicating variation and plasticity in this pathway in arthropods (38). Studies suggest that the IMD pathway is triggered by different pathogens depending on the species and the response generated may even differ between different tissues (164). For instance, in *Eriocheir sinensis* both Gram^-^ (*V. parahemolyticus*) and Gram^+^ (*Staphylococcus aureus* and *Bacillus subtilis*) bacteria can activate the IMD pathway inducing the translocation of Relish to the nucleus (186). Interestingly, all these bacteria upregulated IMD expression and induced translocation of Relish regardless of the type of PGN, DAP-type (*V. parahemolyticus and B. subtilis*) and Lys-type (*S. aureus*) (186). Moreover, the expression of Relish was upregulated in *S. paramamosain* (187), *P. monodon* (173) and *L. vannamei* (128) in response to WSSV infection. Nonetheless, further studies need to confirm if this upregulation of Relish is part of the immune mechanism triggered to abolish this pathogen or part of the WSSV replication mechanism, which has been suggested to hijack this NF-κB pathway to favor its own propagation (128).

Similar to mammals, studies on *L. vannamei* showed that MKK4 can phosphorylate and activate p38 MAKP (175), whereas DmMKK4 exclusively activates JNK in *Drosophila* (188). Additionally, Wang and co-workers proved in *L. vannamei* that TAB1 can combine with TAK1 and p38 MAKP, thus regulating the activity of these kinases (169). Therefore, p38 MAKP regulation in crustaceans can be achieved by three routes: MKK6, MKK4, and TAB1. Consequently, as proposed by Li et al. (66), the presence of different pathways could provide precise control over p38 MAKP activity during pathogenic invasion, indicating the significant involvement of p38 MAKP in crustacean immunity. For instance, even if one or two pathways are blocked under particular conditions, p38 MAKP may still function in the immune response (66).

Until recently, it remained unclear how the IMD pathway can sense pathogens, since no members of PGRP have been described in crustaceans. Besides, homology searches of shrimp (189, 190) and crab (186) transcriptome data failed to uncover a PGRP homolog. Even though the existence of PGRPs remains doubtful in crustaceans, Shi and co-workers (38) suggested a class B2 scavenger receptor (SRB2) as potential receptor of the IMD pathway. Studies conducted in *M. japonicus* showed that SRB2 can sense LPS using its extracellular domain and interact with IMD with its C-terminal intracellular region; thus, amplifying the signal and promoting Relish’s nuclear translocation (38). SRB2 silencing by RNAi increased bacterial load and decreased the survival rate to *V. parahaemolyticus* infection by abolishing the expression of AMPs (38). Therefore, it was concluded that SRB2 stimulates bacterial clearance and enhances shrimp survival rates to Gram^-^ bacterial infection by increasing the transcripts of anti-lipopolysaccharide factors 1 (Alf1) and 2 (Alf2) (38). In this context, the authors also identified homologs of this receptor in other crustacean species such as *L. vannamei*, *P. clarkii*, *Scylla serrata* and *Hyalella azteca*; consequently, confirming the hypothesis that SRB2 could act as signal transduction membrane receptor of the crustacean IMD pathway. Nevertheless, since SRB2 cannot recognize PGN (38) and is thus unable to sense Gram^+^ bacteria, it remains unknown how the IMD pathway is activated after Gram^+^ challenges.

Research into the IMD pathway in crustaceans should focus on several interconnected areas that could enhance our understanding of immune responses. Firstly, the characterization of SRB2 as a potential sensor for LPS (38) invites detailed investigation into its molecular interactions with IMD, as well as the broader implications for AMP expression. Moreover, examining the functional roles of specific AMPs generated through the IMD pathway can elucidate their mechanisms against diverse pathogens, which may involve gene knockout approaches to clarify their individual contributions to immunity. The impact of environmental stressors on the responsiveness of the IMD pathway and AMP production warrants exploration (191, 192), as such factors could significantly influence crustacean health and resilience in aquaculture settings. Integrating these studies with multi-omics approaches, encompassing genomics, transcriptomics, and proteomics, could provide a comprehensive view of the signaling networks and regulatory mechanisms involved in the IMD pathway, potentially revealing novel interactions that facilitate pathogen resistance. Lastly, investigating how pathogens, like WSSV, hijack the IMD pathway (95) could uncover strategies for mitigating their detrimental effects on crustacean populations, enhancing overall disease management in aquaculture.

## 7 Antimicrobial peptides

AMPs can be found in all kingdoms of life and exhibit an extraordinary structural and functional diversity, thus making them possible alternatives to antibiotics (193). This diverse group of peptides are categorized into subgroups or families based on their structural properties, as determined by the peptide’s primary sequence and three-dimensional (3D) conformation. According to these criteria, they can be classified as one of four types, α-helix, β-sheet, extended and β-hairpin or loops. On the other hand, using mechanisms of action, these molecules can be categorized as membrane disruptive or nonmembrane disruptive AMPs. The first creates holes in the membrane whereas the second group pass into cells to act on intracellular targets in order to disrupt metabolism and kill. Either way, these peptides produce a disturbance and disorder of the cytoplasmic membrane resulting in loss of the transmembrane potential, and eventual cell death. This mechanism of action based on membrane interactions rather than through recognition of a single receptor like antibiotics, makes the development of bacterial resistance improbable (194–196). AMPs are produced as the first line of defense against infection, and in addition to their antimicrobial properties these peptides have also been shown to have immunomodulatory functions. Some of these immunostimulant effects include stimulation of chemotaxis, immune cell differentiation, initiation of adaptive immunity and stimulation of both pro- and anti- inflammatory cytokines (197).

Crustacean AMPs are mostly small, amphipathic, cationic, and gene-encoded peptides that are mainly produced by hemocytes or originate from proteins involved in other biological functions (195). There are 15 families that can be grouped, according to Rosa and Barroco (195), into four main groups based on amino acid sequence and 3D conformation. These groups comprise: (I) single-domain linear α-helical AMPs and peptides enriched in certain amino acids, (II) single-domain peptides containing cysteine residues engaged in disulfide bonds, (III) multi-domain or chimeric AMPs, and (IV) unconventional AMPs including multifunctional proteins and protein-derived fragments that exhibit antimicrobial functions (195). Herein only an overview of key crustacean AMPs is provided, for further information on this issue see reviews by (195, 198–200).

Crustins are a family of peptides belonging to group III, characterized by the presence of a whey acidic protein (WAP) domain at the C-terminus (195). Based in their structure, crustins sub-cluster in three types (I, II and III). Type I is characterized by a cysteine-rich region located between the leader sequence and the WAP domain; type II is defined by the presence of a hydrophobic area containing a glycine-rich region upstream of the cysteine-rich region described for Type I crustins; whereas type III have a concise proline/arginine-rich area between the WAP domain and the leader sequence, and do not possess any other amino acid rich domains like type I and II (195). Crustins type II and III are mostly found in shrimp and crayfish, while type I is more exclusive of crab (195). Regarding their function, crustins have been shown to regulate intestinal microbiota balance and have direct antibacterial activity against several bacterial species such as *S. aureus*, *Bacillus* sp., *V. parahaemolyticus*, *Vibrio harveyi*, *Vibrio anguillarum* and *Vibrio alginolyticus* (201). Some studies have shown the modulation of crustin transcripts in infection trials, as well as the deleterious effect of knocking down the transcription of crustin genes on shrimp survival (201). Thus, this AMP play a pivotal role in crustacean immune responses against pathogens.

The anti-lipopolysaccharide factors (ALFs) family is part of group II of crustacean’s AMPs (195). These peptides exhibit a broad spectrum of binding affinities and antimicrobial activities against pathogens. This diversity is a consequence of a significant variation in amino acid sequences and LPS-binding domains (LPS-BD) among members of this family (200). Based on these characteristics, ALFs are clustered in seven groups (Groups A to G) (200). Interestingly, members of groups B, C and F exhibit cationic charge whereas groups A, D, E and G displays anionic properties (200). Additionally, ALFs present a wide range of antimicrobial activities; thus, peptides such as *P. monodon* ALFPm3 displays a strong activity against fungi, virus, and both Gram^-^ and Gram^+^ bacteria, whereas other members exhibit low or insignificant microbicidal properties (200). Consequently, it has been hypothesized that some ALFs may work together with other AMPs or play roles in other biological processes, such as wound healing and tissue regeneration (202). In this context, the potential use of ALFPm3 in aquaculture has been proposed due to its immunostimulant and antimicrobial activities against WSSV, yellow head virus and *V. harveyi* infections (18).

Part of group III of crustacean AMPs, penaeidins and stylicins represent two different families of peptides mostly identified in penaeid shrimp with species-specific function (Reviewed by 189). Currently, only a few members of these families have been described in commercially valuable species such as *L. vannamei, P. monodon, M. japonicus* (200). Thus, further studies need to address the existence and function of these peptides in other crustacean species. So far, it is generally accepted that stylicins are mainly antifungal peptides (200). Meanwhile, penaeidins which subcluster into two subfamilies (I and II) display antimicrobial activities against Gram^+^ bacteria and filamentous fungi for type I; and antibacterial activities against Gram^+^ and Gram^-^ bacteria for type II (18, 200).

Remarkably, besides the typical described AMPs, current evidence supports the key role of other protein-derived peptides in crustacean immunity (200). In this group, AMPs derived from hemocyanin-cleavage are the most well-known. These peptides have been reported for species such as *L. vannamei*, *P. stylirostris*, *P. monodon*, *P. japonicus* and *F. chinensis*, and exhibit a broad range of antimicrobial activities specie-specific (18). In this context, researchers are looking into pleiotropic peptides such as Pituitary Adenylate Cyclase-Activating Polypeptide (PACAP), which has shown immunostimulant and antimicrobial properties in shrimp and crayfish species (203, 204).

Gene encoded AMP expression is linked to NF-κB pathway activation (Reviewed by 33). Notably, in crustaceans, the expression of peptides such as crustins, penaeidins and ALFs have been associated with both Toll and IMD pathways (66). Moreover, recent studies in *P. clarkii* show that during *A. hydrophila* infection, CTLs can also trigger ALFs upregulation via the JNK pathway (57).

As discussed previously, these AMPs serve as one of the first lines of defense against pathogens, showcasing the complexity of immune signaling in invertebrates (195). The coordinated response involving the Toll and IMD pathways not only emphasizes the adaptive nature of crustacean immunity but also allows development of sustainable, antibiotic-free disease management strategies in aquaculture. In this context, given the crucial roles that the Toll and IMD pathways play in regulating AMP expression (66), further research is essential to deepen our understanding of crustacean immune responses. One potential avenue of research could focus on the molecular mechanisms underlying the crosstalk between these pathways and other signaling cascades, such as MAPK pathways, which have been suggested to influence AMP expression (168, 175). Investigating how these pathways interact could reveal novel regulatory networks that govern immune responses in crustaceans.

Additionally, studies could explore the environmental factors that influence the expression of AMPs in crustaceans. Understanding how abiotic factors such as temperature, pH, salinity, and pollution impact the regulation of immune responses could be critical for improving aquaculture practices. Another important direction would involve evaluating the genetic diversity of AMP genes in various crustacean species and their responses to different pathogens, which could inform selective breeding programs aimed at enhancing disease resistance in aquaculture.

Finally, there is a need to investigate the potential for utilizing these AMPs as biocontrol agents in aquaculture. As antibiotic resistance becomes an increasingly pressing issue (10), exploring the application of AMPs in disease management could provide sustainable solutions for improving shrimp health and productivity.

## 8 Environmental stressors influence Crustaceans’ susceptibility to diseases

**Figure 3:**
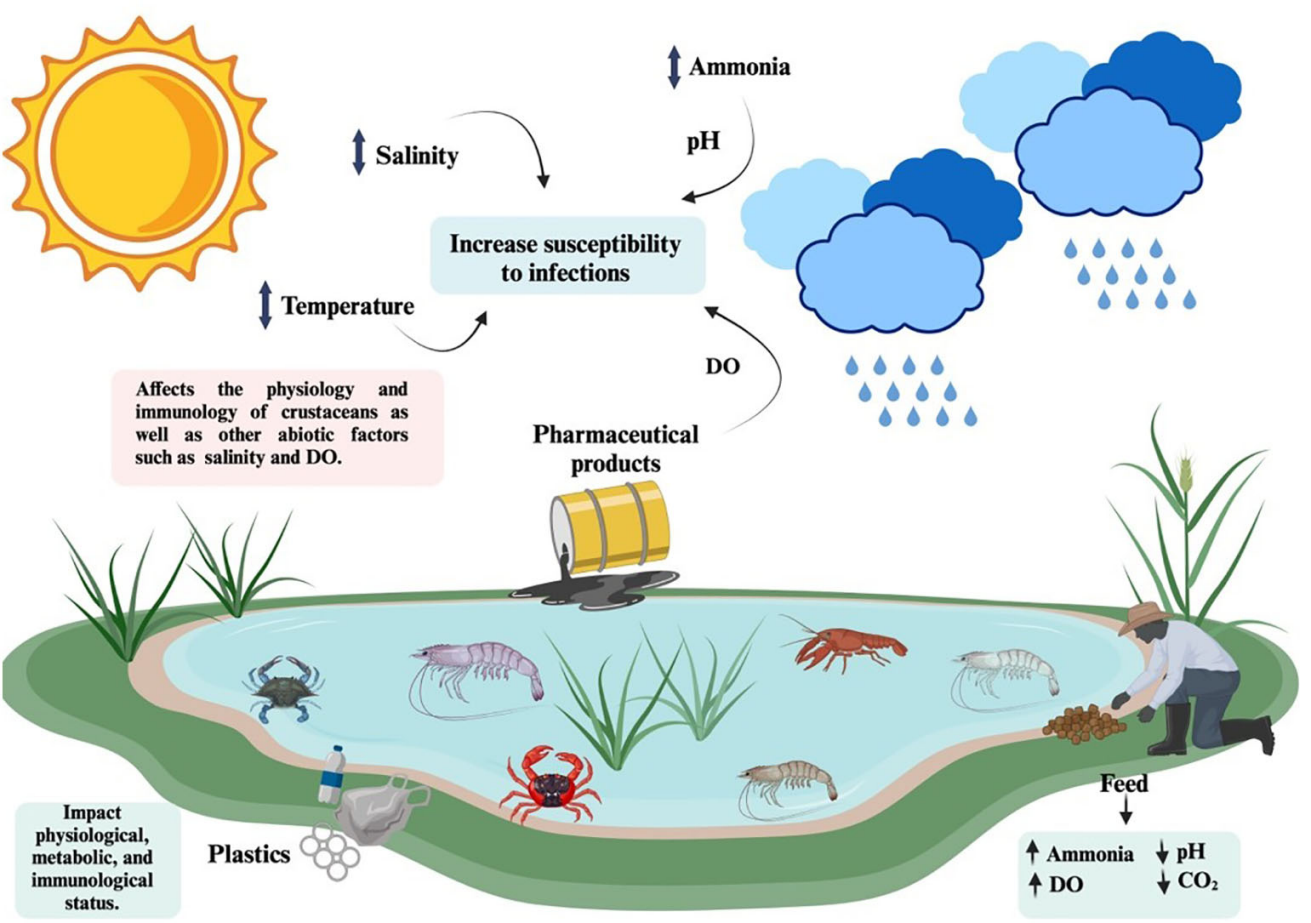
Environmental factors such as temperature, salinity, pH, dissolve oxygen (DO), ammonia concentrations and pharmaceutical products, significantly influence pathogen susceptibility in crustaceans, thus contributing to disease outbreaks in aquaculture facilities. Created in BioRender. Betancourt Aguiar, J. (2024) BioRender.com/n17f466.

Crustaceans are constantly subjected to environmental challenges (**Figure 3**). Extreme temperature fluctuations and environmental contaminants (i.e., plastics and pharmaceuticals) are among the stresses that have a metabolic cost to maintain homeostasis and that can cause a weakening of immune defenses and greater vulnerability to diseases (8). In addition, the rapid growth of crustacean industry and particularly shrimp farming have resulted in environmental pollution affecting farmed and wild aquatic species (205). Pollution may lead to variation in abiotic variables such as temperature, dissolved oxygen, ammonia levels, pH and salinity, affecting health status and crustacean’s innate immune responses to pathogens (206). Genes differentially expressed (DGs) in response to environmental stresses are involved in different signaling pathways including TLR/IMD-NF-κB, JAK-STAT, MAPK, and Wnt signaling pathways, and these pathways are commonly activated simultaneously (207).

Water temperature is probably the most important abiotic variable affecting aquatic species. It affects other abiotic variables such as salinity and oxygenation of the water (208) and may affect survival, growth, physiological functions, and immune defenses (198). Although crustaceans can tolerate a wide range of temperatures, and cultured species are commonly reared in shallow ponds with wide seasonal and daily temperature fluctuations, the effects of these variations on the ability of these species to fight pathogens are not yet fully understood (209). For instance, the thermal maxima (CTMax) and minimum (CTMin) of the most farmed crustacean species in the world, the Pacific white shrimp *L. vannamei* (210), are between 36 and 42°C and between 8 and 12°C, respectively, depending on the life stage, acclimatization temperature and temperature ramp rate (211). However, it’s been shown that the susceptibility of *L. vannamei* to pathogens is highly dependent on the water temperature. A range between 25 and 28°C offers optimal conditions for WSSV infections leading to high shrimp mortalities, while higher temperatures (33–34°C) have shown to be protective (209). Millard and coworkers (209) suggested that elevated temperatures may have an effect on the activity of viral enzymes or on the ability of the host to respond to the pathogen. In one study, WSSV-inoculated shrimp were kept in a four-compartment system with all chambers at 27°C or with a thermal gradient (27–29–31–33°C), and shrimp that were allowed to migrate to warmer temperatures (behavioral fever) showed a significant higher survival rate that shrimp that were kept at 27°C (212). It’s known that warm waters (32-33°C) inhibit WSSV replication (212, 213), but the shrimp defense mechanisms behind the protective effect of elevated temperatures still need more attention from researchers. In this sense, it is suggested that high temperatures activate the unfolded protein response (UPR) protein kinase R-like ER kinase-eIF2α (PERK-eIF2α) signaling pathway, inhibiting the translation of proteins, including WSSV proteins, and inhibiting WSSV proliferation (207). Hyperthermia has also been associated with cell apoptosis in WSSV-infected *L. vannamei* as a mechanism responsible for an increased survival rate (214). Apoptosis as well as endocytosis pathways were involved in the response to heat stress of *Palaemon graviera* (205). In this transcriptomic study acute heat stress induced upregulation of the following immune related-transcripts: caspase 7 (CASP7), transcription factor AP-1 (Jun), broad-complex core protein (BR-C), heat shock proteins 70 (HSP70), Rab5B and Rab10. These authors point out the need of protein level assays to corroborate their results and suggest that acute high temperature may significantly reduce the metabolic capacity of shrimp but enhance the immune capacity, as an emergency metabolic compensation strategy to deal with stress (215).

On the other hand, acute cold-stress (13°C) caused a decrease in plasma metabolite concentrations, relative gene expression related to UPR pathway and apoptosis in the hepatopancreas and hemocytes of *L. vannamei*, with histological damage in the hepatopancreas, suggesting a decrease immunity and more vulnerability to diseases (216). In this context, a study conducted by Wang and coworkers (191) reported a decrease in the gene expression of TLR, IMD and proPO in the intestine of *L. vannamei* after cooling (28°C to 13°C). Thus, the authors argued that considering that the intestinal barrier represents one of the first line of host defenses against invasive microorganisms and that TLRs, IMD and the proPO system play key roles in immune defenses, the reported downregulation of these genes could indicate a reduction in the shrimp’s ability to identify and eradicate pathogens during low temperatures (191). Lv et al. (192) also suggested that unsuitable temperatures have detrimental effects in the gene expression levels of core proteins in the NF-κB signal pathway. Moreover, temperature variations also influence other abiotic parameters such as salinity and oxygenation of the water. It may affect crustaceans immune related variables including clotting times, total hemocyte count, phagocytosis, antibacterial activity of the hemolymph and pro-phenoloxidase activity (208).

Other major abiotic variable affecting the innate immune system of crustaceans is level of ammonia (205). High concentrations of ammonia caused an increased mortality, a decrease in the phagocytic activity and a decrease in the clearance efficiency in *L. vannamei* challenged with *V. alginolyticus* (217). In the same species, high ammonia levels caused a suppression of immune parameters such as a 66% decrease in total hemocyte count (218), an increased coagulation time and a down-regulation in transglutaminase gene expression (219). In this line, studies conducted in *P. monodon* indicate that ammonia exposure seems to initially trigger an immune response, but over time, prolonged stress weakens the immune system. This overexposure leads to reduced immune enzyme activity and downregulates the expression of immune-related genes like lysosome and crustin (220). Moreover, ammonia stress downregulates CTLs expression in *P. monodon’s* hepatopancreas and intestine (221, 222); thus, considering that CTLs play an important role as PRPs during *V. harveyi* and *V. anguillarum* challenges (222), high concentrations of ammonia could increase the vulnerability of shrimp to bacterial diseases. CTLs have also shown to act as critical immune components against *V. parahaemolyticus* and WSSV infections, and during endoplasmic reticulum (ER)-stress responses in *L. vannamei* (223).

Environmental contaminants including plastics and pharmaceutical products are constantly discharged into aquatic environments affecting aquatic wildlife. Chronic toxicity of chemical contaminants in crustaceans has shown to be dependent on abiotic variables such as temperature. In one study, the negative impact of clofibric acid (CA) and diclofenac sodium (DS) on *Palaemon longirostris* larvae development was significantly higher at 18°C compared to 24°C (224). Plastics and especially microplastics (MPs) and nanoplastics (NPs) are aquatic contaminants that accumulate in tissues of aquatic organisms affecting their immunity and metabolism (225). Exposure to MPs caused variation in the intestinal microbiota of *L. vannamei* inducing the proliferation of opportunistic pathogens in the gut. Different MPs changed the hemolymph proteomic composition, specifically proteins involved in inflammation, apoptosis, oxidative stress and metabolism (212). MPs and NPs also caused physical damage to intestinal cells including epithelial cell necrosis (226) and shorter intestinal fold heights (227). More studies are needed to understand the impact of micro and nanoplastics on the health and immune response of crustaceans.

As previously stated, JNK and p38 MAPK are also referred as SAPKs because their role in transmitting environmental stress signals to the nucleus (159). Zheng and coworkers (228) described in *L. vannamei* that under low temperature stress JNK plays a crucial role regulating apoptosis and oxidative damage. JNK is activated by excessive reactive oxygen species (ROS) production and accumulation caused by an increase in the mitochondrial respiratory rate induced by low temperature (228). Activated JNK activates AP-1 transcription factors, thus leading to the regulated expression of downstream apoptosis genes such as p53, caspase-3 and mitochondrial proteins associated with apoptosis such as Bax and Bid (228). However, the expression of some of these apoptosis related genes was also significantly enhanced after JNK silencing; furthermore, ROS accumulation, apoptosis and mortality erratically increased after this silencing. Thus, the authors proposed that JNK is essential for mediating low temperature tolerance, apoptosis rate and ROS accumulation (228). Also, Tian et al. (229) described that molt could trigger apoptosis driven by oxidative stress through the activation of JNK in *P. clarkii*. Consequently, JNK could be mediating this process by inducing the expression of pro-apoptotic genes such as Bax, Bak and Bok, and inhibiting the expression of anti-apoptotic genes such as Bcl, A1 and Mcl1 (229).

Luo and coworkers (230) reported that acute cold stress activates the gene expression and phosphorylation of p38 MAPK, in gill tissues of *L. vannamei*, suggesting its potential role in response to low temperatures. Interestingly, although the gene expression levels of p38 MAPK, JNK and ERK were measured, only the p38 MAPK values were upregulated during the first 12 h of cold stress exposure (230). Consequently, these findings contradict the upregulation of JNK associated with ROS production induced by low temperatures as described by (228) in *L. vannamei* hemocytes. Accordingly, Luo et al. (230) results could be interpreted in light of the non-significant production of ROS in the gill during the timeframe of the experiment, which may have limited the expression of JNK; or the central role of p38 MAPK responding to this environmental stressor in the gill instead of JNK. However, further studies are necessary to clarify these observations by analyzing the expression levels of the MAPKs as well as other signaling proteins involved on these pathways, alongside apoptosis-related genes. Additionally, measuring ROS production across multiple tissues under low-temperature stress would help clarify the mechanisms at play.

A study conducted by Park et al. (231) in *M. japonicus* illustrated that p38 MAPK plays a key role mediating the immune response against oxidative stress induced by environmental pollutants such as perfluorooctane sulfonate (PFOS), irgarol, di(2-ethylhexyl) phthalate (DEHP), and bisphenol A (BPA). In this study significant expression of p38 MAPK was mainly detected in the gill and hepatopancreas tissues (231), suggesting their pivotal role during toxic pollutants exposure. The authors suggested that p38 MAPK could be orchestrating inflammation, apoptosis, and cell cycle regulation processes in response to these pollutants (231). Nevertheless, additional research is required to understand these issues.

Thus, Shui and coworkers (232) proposed that p38 MAPK orchestrate the distribution of cadmium (Cd) in *P. clarkii* crayfish by modulating the accumulation of Cd in different tissues under Cd stress environments. Results suggest that a strong negative correlation exists between Cd levels and the expression of p38 MAPK in several tissues. Moreover, p38 MAPK transcript levels showed relevant differences between tissues and under no stress versus stress conditions (232). The tissue-specific differences in p38 MAPK gene expression in the absence of Cd exposure, compared to conditions of low and high exposure, indicates that crayfish absorb Cd through their gills, after which the absorbed Cd is distributed to various tissues, including the hepatopancreas, heart, antennal gland, and muscle. However, the primary site of Cd accumulation is regulated by the p38 MAPK pathway in the hepatopancreas, which serves as the main Cd storage tissue in *P. clarkii* (232). Given this context, while the modulation of Cd tissue distribution by p38 MAPK is intriguing, further research is necessary to identify the specific genes targeted by this MAPK signaling pathway that facilitate this outcome. Probably, these genes are involved in regulating ion and osmotic channels within the cells, which may play a crucial role in the absorption and accumulation of Cd in various tissues. Furthermore, it is particularly interesting that a study carried out by Jian et al. (233) in *S. paramamosain* reported a significant increase in one of the three isoforms of IKK described for this specie after 6h of Cd exposure. Therefore, the IKK complex, which is considered one of the core elements of the NF-κB cascade, is also involved in coping with Cd pollution (233). Thus, a better understanding of these mechanisms could provide valuable insights into how crustaceans manage Cd stress and other environmental contaminants.

It is widely recognized that environmental stressors significantly influence pathogen susceptibility in crustaceans, which is a primary contributor to disease outbreaks in aquaculture facilities. Such stressors can compromise the immune response of crustaceans, thereby increasing their vulnerability to pathogens and result in substantial economic losses within the aquaculture sector (11, 209). As highlighted in the preceding sections, variations on temperature influence susceptibility to WSSV infections (212, 213) as well as the prevalence of *Vibrio* infections (234). Moreover, ammonia accumulation on farms, resulting from the continuous influx of biological waste, residual bait, and crustacean exoskeletons, can trigger and amplifies infections by *V. alginolyticus* (217), WSSV (235) and *Lactococcus garvieae* (236). Furthermore, low-salinity stress also enhances susceptibility to infections by *V. alginolyticus* (237) *L. garvieae* (236)*, V. harveyi* (238)*, V. parahaemolyticus* (234) *and Vibrio cholerae* (234) [see also reviews by (209, 238)]. Ultimately, while the crustacean immune system has evolved to effectively counter pathogens, environmental stressors can induce extreme conditions that adversely affect their physiology and immune responses. As survival becomes the primary focus under these conditions, crustaceans become increasingly susceptible to pathogenic infections. Thus, comprehension of the interactions among environmental stressors, pathogens, and host is essential for developing effective management strategies to reduce disease risks in aquatic ecosystems.

Despite the numerous publications describing changes on crustaceans’ immunity and health indicators due to environmental stressors, more evidence is needed to understand the effects of these changes during an encounter with a pathogen (8) and the variability of the responses depending on different stressors (209). Additionally, a comprehensive understanding of how crustaceans perceive abiotic variations, the signaling pathways involved, and the differential gene expression that these stressors trigger is essential for grasping the variability of immune responses. Furthermore, stressors such as temperature variations and environmental contaminants not only affect the immune functions, of both farmed and wild shellfish, but also have serious implications for toxin accumulation, necrotic tissue, muscle atrophy, changes in organoleptic profiles and discoloration (8). Thus, gaining deeper insights into these mechanisms will be crucial for developing effective management strategies to enhance the health and resilience of crustacean populations in both aquaculture and natural environments.

## 9 Concluding remarks

Living in a hostile and microbe-enriched environment, crustaceans exhibit a sophisticated and highly effective immune system, which has assured their survival and expansion across evolution. A continuous environmental pathogenic pressure as well as abiotic and biotic stressors have driven to the refinement of their immune system (11). Therefore, this group of invertebrates exhibit unique features that are highly relevant for understanding the immune mechanism underneath, which differ from the traditional invertebrate model and open the possibility of reaching new insights and paradigms in immunology.

A suit of PRR have been characterized in crustacean, thus empowering them with a broad spectrum of pathogen recognition. Interestingly, the role of some proteins such as HDL [also known as βGBP in penaeids (27, 28)] and hemocyanin (18) which have been described involved in the recognition of pathogens and triggering immune responses, respectively, bring to focus the close relationship between the crustacean physiological and immunological status; as well as provide evidence of a potential evolutionary adaptation process that these organisms undergo toward multitasking optimization. In this context, crustacean TLRs have the unique ability of triggering a defensive response either through direct recognition of PAMPs or via Spz-mediated activation, thus providing them with a refined pathogen recognition mechanism. Additionally, this defense system benefits from the ability to respond to both Gram^+^ and Gram^-^ bacteria through the Toll and IMD pathways (66). In this line, the IMD pathway displays the singular characteristic of recognizing Gram^-^ bacteria through the SRB2 as consequence of the absence of PGRP in crustaceans (38). Moreover, this pathway finely regulates p38 MAPK through MKK6, MKK4, and TAB1 proteins, highlighting its crucial role in immune response development during pathogenic invasion (66).

Environmental stressors significantly shape the immune responses of crustaceans by modulating key signaling pathways, including NF-κB and MAPKs (228, 232, 233). For instance, temperature variations affect the TLR and IMD gene expression levels as well as the proPO activating-system (191). Meanwhile, CTLs transcripts are vulnerable to ammonia exposure (222), thus disturbing the recognition of pathogens. Moreover, fluctuating temperatures and exposure to pollutants can alter the expression and activity of crucial proteins like JNK (228) and p38 MAPK (230, 232), which play key roles in transmitting environmental stress signals to the cell nucleus (159). For example, in *L. vannamei*, JNK is activated by ROS production due to elevated mitochondrial respiratory rates under low-temperature stress (228). This activation leads to the expression of pro-apoptotic genes, indicating that JNK is crucial for mediating low-temperature tolerance and apoptosis (228). In contrast, while p38 MAPK is also activated under cold stress (230), its role in regulating immune responses against environmental pollutants, such PFOS, DEHP, BPA and Cd, has been highlighted in various studies (231, 232). These findings underscore the need for further investigation to clarify the interplay between these signaling pathways and their associated genes in response to abiotic stressors.

Overall, crustaceans portray unique immunological features, that further knowledge of could lead to new disease management strategies in aquaculture. For instance, AMPs are part of the prominent immune responses triggered against pathogens in crustacean and constitute potential candidate for the development of new and effective therapies that can improve crustaceans’ health and responses to diseases in aquaculture. Moreover, farmers could benefit of a better understanding of the environmental stressors affecting crustacean aquaculture, since these factors are the primary cause of the disruption of the balance between host and pathogens which lead to disease outbreaks. Finally, the potential for immunological memory in crustaceans remains an area of active investigation, with most studies linking their findings to immune priming and trained immunity responses (239). However, much of the current research has focused primarily on describing immunological changes through survival rates in animals challenged with homologous pathogens (62, 239). To advance understanding, further research is needed to explore the underlying immunological pathways and metabolic alterations in innate immune cells, particularly regarding chromatin remodeling—a key distinction between immune priming and trained immunity, with the latter offering more prolonged protection (240). In this context, it is crucial to determine whether the epigenetic changes associated with trained immunity can enhance the expression of immune genes, such as PRPs and AMPs, thereby enabling more precise immune recognition and response to pathogens.
